# Transfection of Sponge Cells and Intracellular Localization of Cancer-Related MYC, RRAS2, and DRG1 Proteins

**DOI:** 10.3390/md21020119

**Published:** 2023-02-10

**Authors:** Kristina Dominko, Antea Talajić, Martina Radić, Nikolina Škrobot Vidaček, Kristian Vlahoviček, Maja Herak Bosnar, Helena Ćetković

**Affiliations:** 1 Division of Molecular Biology, Ruđer Bošković Institute, 10000 Zagreb, Croatia; 2 Division of Molecular Medicine, Ruđer Bošković Institute, 10000 Zagreb, Croatia; 3 Department for BioMedical Research, University of Bern, 3008 Bern, Switzerland; 4 Bioinformatics Group, Molecular Biology Department, Division of Biology, Faculty of Science, University of Zagreb, 10000 Zagreb, Croatia

**Keywords:** Porifera, transfection, DRG1, MYC, RRAS2, primary sponge cells, intracellular localization

## Abstract

The determination of the protein’s intracellular localization is essential for understanding its biological function. Protein localization studies are mainly performed on primary and secondary vertebrate cell lines for which most protocols have been optimized. In spite of experimental difficulties, studies on invertebrate cells, including basal Metazoa, have greatly advanced. In recent years, the interest in studying human diseases from an evolutionary perspective has significantly increased. Sponges, placed at the base of the animal tree, are simple animals without true tissues and organs but with a complex genome containing many genes whose human homologs have been implicated in human diseases, including cancer. Therefore, sponges are an innovative model for elucidating the fundamental role of the proteins involved in cancer. In this study, we overexpressed human cancer-related proteins and their sponge homologs in human cancer cells, human fibroblasts, and sponge cells. We demonstrated that human and sponge MYC proteins localize in the nucleus, the RRAS2 in the plasma membrane, the membranes of the endolysosomal vesicles, and the DRG1 in the cell’s cytosol. Despite the very low transfection efficiency of sponge cells, we observed an identical localization of human proteins and their sponge homologs, indicating their similar cellular functions.

## 1. Introduction

Transfection is a procedure that introduces foreign nucleic acids into eukaryotic cells, modifying them genetically. Since the 1980s, transfection has been an important method in basic scientific research, the pharmaceutical industry, and medical uses, including the production of mRNAs, recombinant proteins in mammalian cells, and biopharmaceutical products (e.g., vaccines). It is one of the most important procedures for molecular cloning, gene manipulation, and gene expression and regulation, as well as protein function and regulation studies. Transfection can be performed biologically (using a viral vector, and then it is called transduction), physically/mechanically (e.g., gene gun, particle bombardment, microinjection, and laser-based transfection), chemically (e.g., calcium phosphate precipitation, cationic polymers, and lipid-based transfection, known as lipofection), or combined (e.g., electroporation) [1,2]. Although there is constant development and progress in transfection techniques, each method has its own advantages and disadvantages, depending on the experimental goal, cell, or tissue type. Herein, we will focus only on the methods used in this study—lipofection and electroporation. Liposome-mediated transfection (lipofection) was first reported by Felgner et al. [3]. The method is based on the formation of liposome complexes that sequester DNA with 100% efficiency [4,5] and is an efficient method for gene transfer both in vivo and in vitro. The lipofection method involves three steps: (1) the formation of complexes between the positively charged cationic liposomes and the negatively charged DNA molecule containing the gene to be inserted; (2) the fusion of the lipid/vector complex with the cell membrane by which the vector is delivered into the cell; (3) the migration of the vector through the cytoplasm and its transport through the nuclear membrane into the nucleus, where it is transcribed with the rest of the cellular DNA [6,7]. The lipid/vector complex is able to enhance gene delivery in different cell types, tissues, and even in combination with viral particles. In addition, lipofection has been used to deliver genes into various tissues in vivo, including lung, endothelium, and muscle, as well as for drug and vaccine delivery. However, precise optimization is required for optimal results, depending on the cell type (adherent cells vs. cells in suspension) or the individual cell line. For example, the presence or absence of serum in the cell culture medium and the ratio of liposome-to-DNA to avoid toxicity are important factors that need to be tested and evaluated before the actual experiment [6]. Electroporation induces the formation of nanometer-size reparative pores in cell membranes by exposing cells to a brief high-voltage electric field and introducing nucleic acids into cells through these pores [1]. Although this method might alter the cell phenotype, it is useful for transfecting a large number of cells at once and often results in higher transfection efficiency and lower cell death, especially within cells that are generally resistant to other transfection methods [4,8].

Transfection by plasmid DNA can be performed in different cell types, including normal mammalian and cancer cells. However, the transfection efficiency varies significantly among cell lines used in experimental research, and some cell lines are not suitable for these types of experiments due to their low transfection efficiency [9]. Fibroblasts are quite difficult to transfect and require significant optimization of the transfection protocol to achieve high cell viability and transfection efficiency. Since dermal human fibroblasts can be easily isolated from the skin or foreskin and easily maintained in cell cultures, they are often used in a variety of biological and medical research, including gene therapy, hereditary diseases, carcinogenesis, and senescence. The optimization of cultivation conditions includes cell density, viability, and appropriate population doublings prior to transfection, as well as the choice of the appropriate transfection method. Lipid-based transfection reagents are commonly used for the transfection of human fibroblast but with different efficiency depending on the reagent used [10]. Another method commonly used for the transfection of “difficult to transfect” cell types, including fibroblasts, is electroporation [8,10]. Standardized cell lines derived from human or nonhuman species (e.g., the Chinese hamster ovary (CHO), HeLa, and human umbilical vein endothelial cells (HUVEC)) are thoroughly characterized and easier to set up for transfection [11]. HeLa cells are the first and probably the most often used cell line. They are extremely malignant, even when compared to other cancer cells from malignant sources. HeLa cells are considered immortal and do not experience cell death after a set number of cell divisions. Importantly, the HeLa cell line is easy to transfect by any method [12]. In addition to many human cell lines, there are numerous commonly used cell lines from other vertebrates, mostly mammals. Transfection is also efficient in other vertebrate cell lines and cells, for example, by the microinjection of foreign DNA into *Xenopus laevis* oocytes [13,14]. In addition, an efficient transfection protocol was developed for the zebrafish cell lines, Pac2, ZF4, and ZFL, by chemical transfection [15,16,17,18,19,20] or cassette-based transfection [21]. Although there are fewer cell lines derived from different invertebrate taxa than vertebrates, there are more than 500 cell lines derived from insects. Hence, most data on the transfection of invertebrates come from insect cells. For *Drosophila melanogaster*, a transfection system that allows the selection of cells with a single-copy transgene inserted at a specific genomic site in S2 cell lines [22] or chemical transfection using either lipofection [23] or calcium phosphate was developed [24,25,26]. In addition, electroporation has been used for the transfection of the corn earworm (*Helicoverpa zea)* and fly (*Musca* sp.) embryos [27] and in the fall armyworm (*Spodoptera frugiperda*) cell line. Lipofection was used for the transfection of the domestic silk moth (*Bombyx mori*), Bm5, fall armyworm (*S. frugiperda*), Sf21, and the spongy moth (*Lymantria dispar*), IPLB LdEp, IPLB-LdEIta, and IPLB-Ld652Y, cell lines [4,5,28,29,30], while calcium phosphate was used for transfection of corn earworm (*H. zea*) cell lines [31,32].

This rapid growth in the number of cell lines produced by terrestrial invertebrates does not reflect the current situation with marine invertebrates. Marine invertebrate primary cell cultures have been used to assess disease in important aquaculture taxa (e.g., crustaceans and bivalves), explore diverse and/or novel cell types, and understand symbioses [33,34,35,36,37]. Despite their obvious value in unraveling complex cellular phenomena, cell lines from most invertebrate taxa are lacking or remain understudied. Significant improvement is needed in the development of invertebrate cell lines, especially in marine invertebrates [38,39]. Successful transfection of crayfish hematopoietic tissue (Hpt) primary cell cultures by electroporation and the efficient expression of foreign genes have been shown [40]. Furthermore, *Crassostrea virginica* hemocytes were chemically transfected using dendrimers, albeit with low transfection efficiency [41]. Moreover, efforts are also underway to develop transfection in basal Metazoa. Previously, the primary cells of Hydra have been successfully transfected using electroporation [42,43,44,45] or a particle gun [46]. Transfection has already been successfully developed in some of the closest unicellular relatives to animals: choanoflagellates, the ichthyosporean *Creolimax fragrantissima* [47], and filasterean *Capsaspora owczarzaki* [48], as well as in amoeba *Dictyostelium discoideum,* by the calcium phosphate method [49,50]. Previously, efforts have been made to develop cell lines from sponges and optimize protocols for the transfection of sponge cells, but with limited success. To date, studies have reported transfection in marine demosponge species [51,52,53,54] or in the homoscleromorph sponge, *Oscarella lobularis* [55]. Unfortunately, the transfections either failed or their efficiency was too low. The major difficulty probably lies in the fact that most marine sponges tolerate only small changes in salinity [55]. However, transfection in the freshwater demosponge species, *Spongilla lacustris* [56] and *Ephydatia muelleri*, also did not show the necessary efficiency and reproducibility [57].

Sponges are ancient animals and possibly the earliest branching animal phylum that has changed little over the last 800 million years [58]. Therefore, they provide important insights into the genomic and proteomic features of the last common ancestor of metazoans [59,60,61]. Despite their simple morphology, sponges have complex genomes [60] with many genes very similar to their vertebrate homologs [59]. Many of these homologs have been linked to human diseases, including cancer and autoimmune diseases [62]. Because no systematic studies have been conducted to clarify the occurrence of tumors in invertebrates, current knowledge about cancer and cancer-related genes in invertebrates is scarce. Based on the available data, we can only speculate about the possible role of cancer-related genes during early animal evolution. A major obstacle to isolating and producing novel compounds from sponges and using sponges to elucidate the physiological roles of genes/proteins involved in human disease is the lack of a large, reliable source of sponge material [51]. The broader interest behind our work is defining characteristics and functions of evolutionarily conserved sponge genes/proteins, with a focus on homologs related to human diseases, particularly cancer. Previous research has shown that these proteins display a high similarity to homologs in “higher” metazoans, not only in primary but also in predicted secondary and tertiary structures, suggesting similar or identical biochemical and biological functions [59,63,64,65,66,67,68,69]. Although we have some insights into the biological function of sponge proteins, the evolutionary conservation of their function in sponges and humans is still poorly understood [70,71,72,73]. Due to the compartmentalization of the eukaryotic cell, the determination of the protein’s intracellular localization is the first step for the understanding of its activation state, interaction partners, correct chemical environment (e.g. a low pH in lysosomes), and finally, biological function [74]. Moreover, in order to regulate protein activity, many biological processes involve changes in the protein’s subcellular localization [75]. All this highlights the importance of studying the development of sponge cell cultures and/or methods for the introduction of DNA into sponge cells in order to study the function of proteins and compounds of interest.

In this study, our first aim was to increase the transfection efficiency of sponge cells from *Eunapius subterraneus*. However, our main focus was to compare the protein localization in the sponge cells with their localization in normal and tumor human cells, a feature that represents the first step in elucidating the function of the protein of interest. Because the focus of our research is to study genes associated with cancer from an evolutionary perspective, we used various methods of DNA transfection to be able to deliver the cDNA coding for sponge and human protein homologs MYC, RRAS2, and DRG1, which are known to be localized to specific compartments (the nucleus, membranes, or cytosol, respectively) and associated with cancer.

## 2. Results

**Figure 1:**
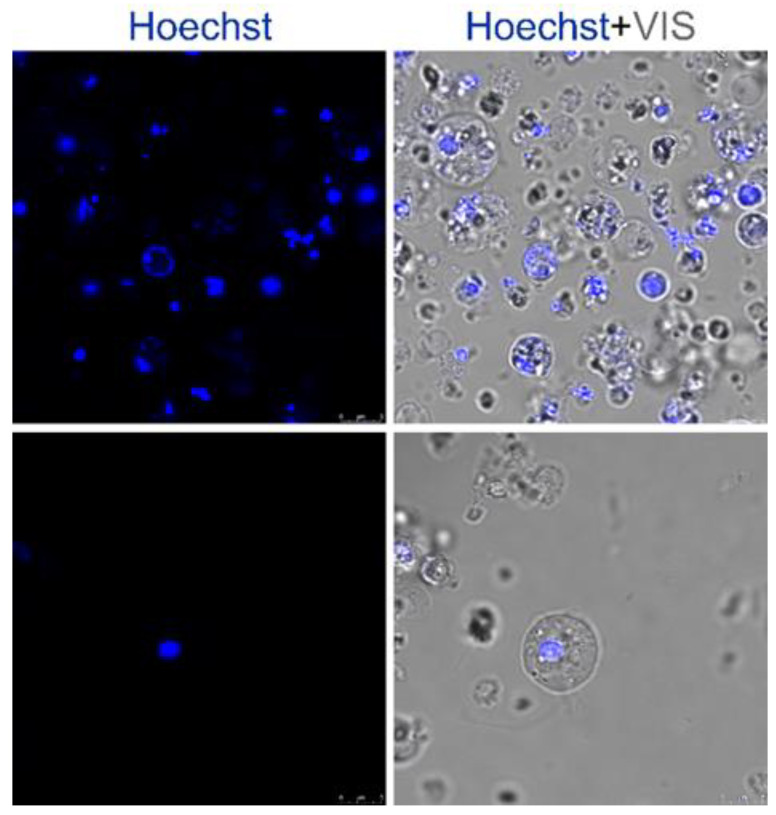
Heterogenous cell culture prepared from the sponge, *Eunapius subterraneus*. The nuclei were counterstained with Hoechst.

To analyze the localization of sponge proteins and for comparing it with the localization of human homologs in tumor and normal human cells, as well as in sponge cells, cDNAs of the genes of interest were cloned into commercially available vectors. Besides the routinely used cancer cell line, HeLa, and the human fibroblast cell line, a primary cell culture of the sponge *E. subterraneus* was also transfected with the same vectors (Figure 1).

### 2.1. Nuclear Protein Localization

The *myc* oncogene (mostly referred to as *c-myc*) was first discovered through its homology with the highly oncogenic retroviral transforming gene (*v-myc*). The protein product of *c-myc* is a transcription factor primarily localized in the cell nucleus regulating fundamental cellular processes, including growth, proliferation, and apoptosis [76,77]. The expression of *c-myc* is tightly regulated in normal conditions, while its dysregulation leads to enhanced levels of MYC, which contribute to tumorigenesis [78]. Members of the MYC family consist of six conserved boxes (MB0, MBI, MBII, MBIIIa, MBIIIb, and MBIV), named Myc homology boxes, the nuclear localization sequence (NLS), and the C-terminal bHLH-Zip domain (helix-loop-helix, leucine zipper) [77,79,80].

**Figure 2:**
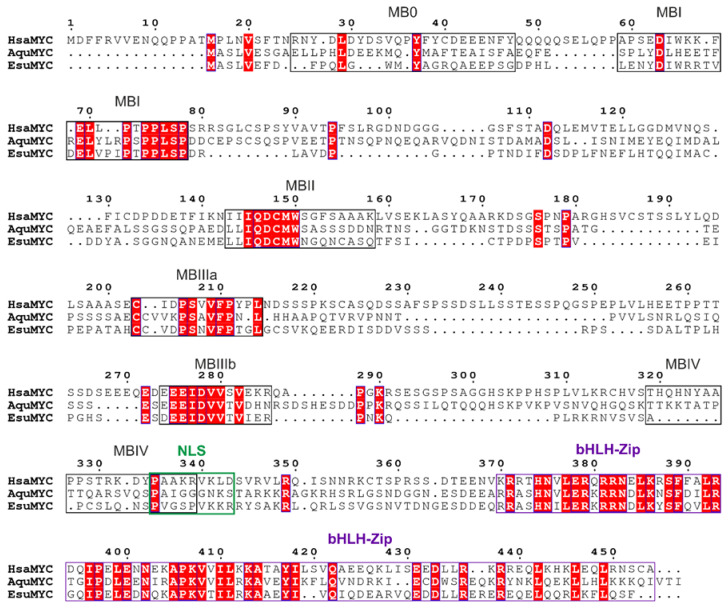
Sequence alignment of sponge and human MYC homologs. The amino acid sequences were aligned using ClustalX 2.0 [81] and visualized using ESPript 3.0 [82]. Conserved domains are marked above the alignment as follows: Six MB boxes are indicated in black, the nuclear localization sequence in green, and the bHLH-Zip domain in purple. Blue frames indicate conserved residues, and white letters in red boxes represent a strict sequence identity.

We have identified MYC homologs from the sponges *E. subterraneus* (accession number OQ148362) and *Amphimedon queenslandica* (accession number XP_003390966.1). An analysis of the protein sequence identity/similarity among the homologs from the sponges *A. queenslandica* or *E. subterraneus* and human (accession number NP_002458.2) revealed that the MYC protein sequence is not highly conserved (Figure 2). The homologs from *A. queenslandica* and *E. subterraneus* showed a 43.2% and 42.1% similarity with the human MYC. The highest homology was found in the bHLH-Zip domain responsible for sequence-specific DNA binding and dimerization, with its binding partner Max, which indicates that the last common ancestor of humans and sponges had a bHLH-Zip domain with basic biochemical properties.

**Figure 3:**
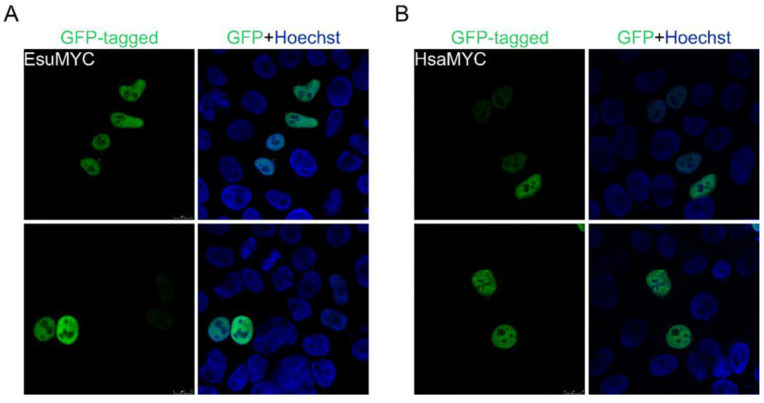
The sponge and human MYC homologs localize in the nucleus of HeLa cells. (**A**) Sponge and (**B**) human MYC were labeled with GFP. Cell nuclei were stained with Hoechst dye. Abbreviations: Esu, sponge *E. subterraneus*; Hsa, human.

**Figure 4:**
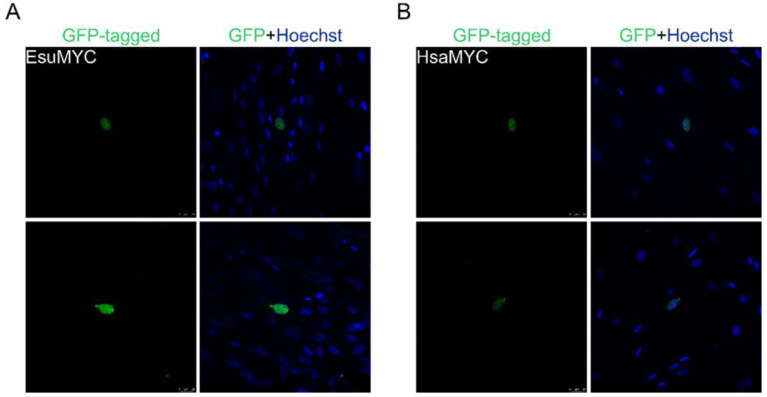
Both sponge and human MYC homologs are localized in the nucleus of human fibroblasts, MJ90. (**A**) Sponge and (**B**) human MYC were labeled with GFP. Cell nuclei were stained with Hoechst dye. Abbreviations: Esu, sponge *E. subterraneus*; Hsa, human.

To analyze MYC protein localization, we cloned cDNA for the sponge and human homolog into a GFP-tagged vector. The analysis of the intracellular localization of the MYC homologs in HeLa cells showed the complete colocalization of the sponge (Figure 3A) and the human MYC protein (Figure 3B) with the Hoechst dye depicting the cell nucleus. Our results confirm the localization of the sponge and human proteins in the nucleus of the HeLa cells but not in the cytosol (Figure 3). Additionally, we analyzed the localization of the sponge and human MYC homologs in the fibroblasts, where we also observed their localization in the nucleus (Figure 4A,B).

**Figure 5:**
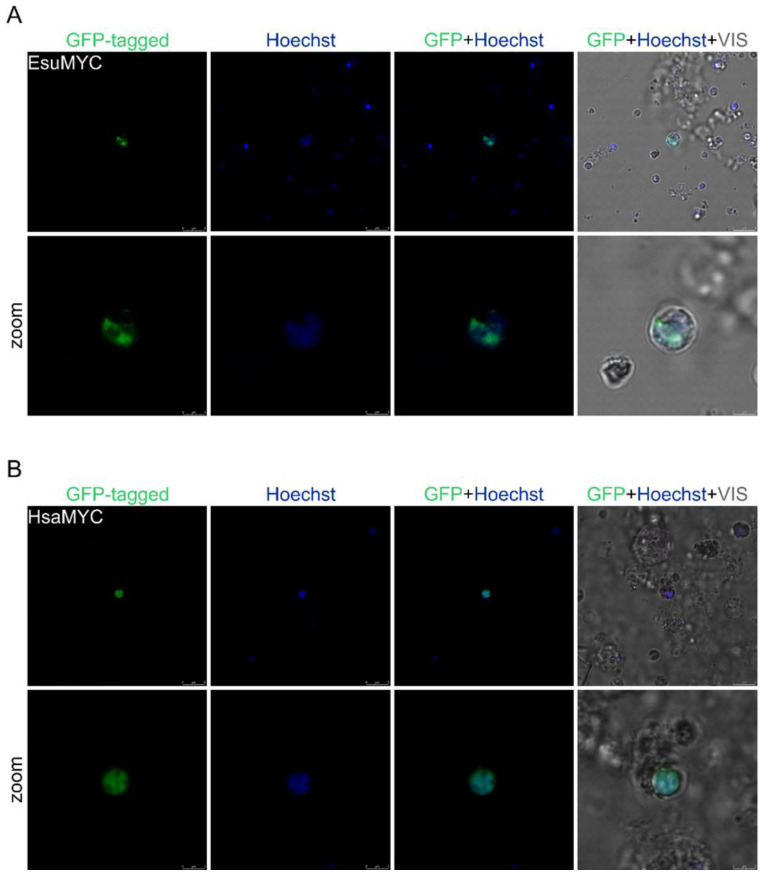
Human and sponge MYC homologs localize in the nuclei of sponge cells. (**A**) Sponge and (**B**) human MYC are labeled with GFP. Cell nuclei were marked with Hoechst dye. Abbreviations: Esu, sponge *E. subterraneus*; Hsa, human.

The localization analysis of the human and sponge MYC proteins revealed that both colocalize with the Hoechst, indicating the nuclear localization of both proteins within the sponge cell (Figure 5A,B). These results demonstrate the identical localization of sponge and human MYC proteins in the nucleus of the sponge and both normal and tumor human cells.

### 2.2. Membrane Protein Localization

**Figure 6:**
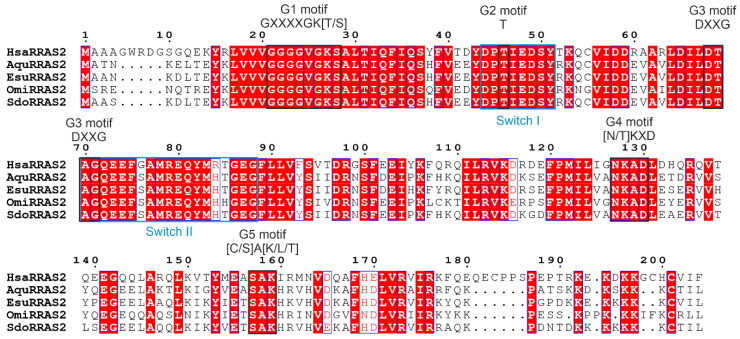
Sequence alignment of sponge and human RRAS2 homologs. The amino acid sequences were aligned using ClustalX 2.0 [81] and visualized using ESPript 3.0 [82]. Conserved domains are marked as follows: Five G-motifs are indicated in black above the alignment, and two switch regions are in light blue under the alignment. Blue frames indicate conserved residues, whereas white letters in red boxes represent a strict sequence identity.

RRAS2 (TC21), a member of the Ras family of proteins, is a small GTPase with an important role in signal transduction that controls multiple cellular processes [83]. The functional dysregulation of RRAS2 has been shown to contribute to oncogenesis, as it triggers critical biological processes in cancer cells, including proliferation [84,85], migration, the epithelial–mesenchymal transition [86], and resistance to chemotherapy [87,88]. RRAS2 is mostly localized in the plasma membrane but also in the Golgi apparatus in HE393T cells [89,90]. We aligned the RRAS2 homologs from sponges *A. queenslandica* (accession number XP_003385162.1), *E. subterraneus* (accession number OQ148363), *Oopsacas minuta* (accession number KAI6654197.1), *S. domuncula* (accession number CAA77070.1), and human (accession number NP_036382.2) in order to analyze the protein sequence identity/similarity (Figure 6). The RRAS2 proteins from the sponges displayed a high homology (a 75–78.4% similarity) with their human homolog. We observed high conservation of the Ras domains (five G-motifs and two switch regions) important for GTPase activity among the sponge and human homologs.

**Figure 7:**
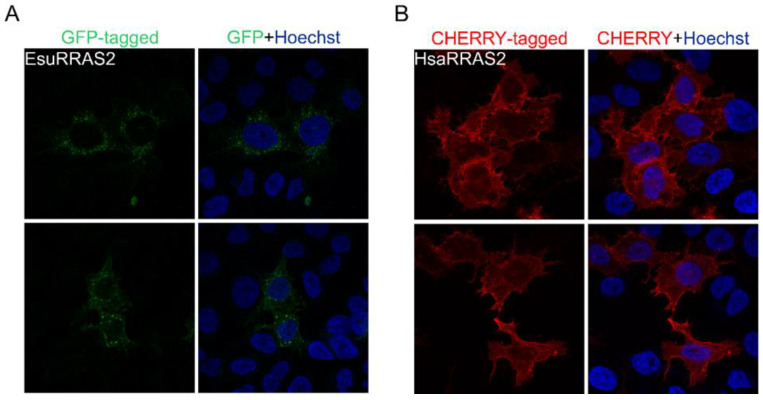
The sponge and human RRAS2 homologs localize in the plasma membrane and cytosolic vesicles of HeLa cells. (**A**) Sponge RRAS2 was labeled with GFP and (**B**) human RRAS2 with CHERRY. Cell nuclei were labeled with Hoechst. Abbreviations: Esu, sponge *E. subterraneus*; Hsa, human.

**Figure 8:**
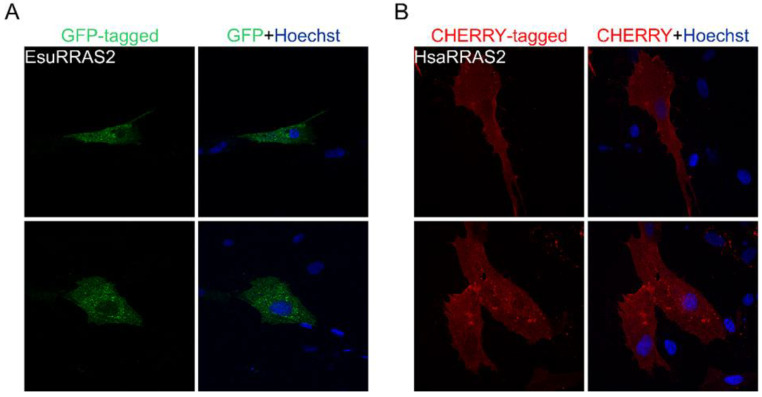
Sponge and human RRAS2 homologs localize in the vesicular membranes and plasma membrane of the human fibroblast cell line MJ90. (**A**) Sponge RRAS2 was labeled with GFP and (**B**) human RRAS2 with CHERRY. Cell nuclei were stained with Hoechst. Abbreviations: Esu, sponge *E. subterraneus*; Hsa, human.

To determine the RRAS2 protein localization, sponge RRAS2 cDNA was cloned into a GFP-tagged and human RRAS2 cDNA into a CHERRY-tagged vector. We observed that the sponge (Figure 7A) and the human (Figure 7B) homolog localize in the cytosol of the HeLa cells but not in the nucleus. In addition, the punctuate staining of the sponge and human RRAS2 indicate their localization in the plasma membrane and vesicular membranes, presumably the endocytic vesicles (Figure 7). Similarly, the sponge (Figure 8A) and human RRAS2 (Figure 8B) proteins were localized in the plasma membrane and membranes of the cytosolic vesicles in the fibroblasts (Figure 8).

**Figure 9:**
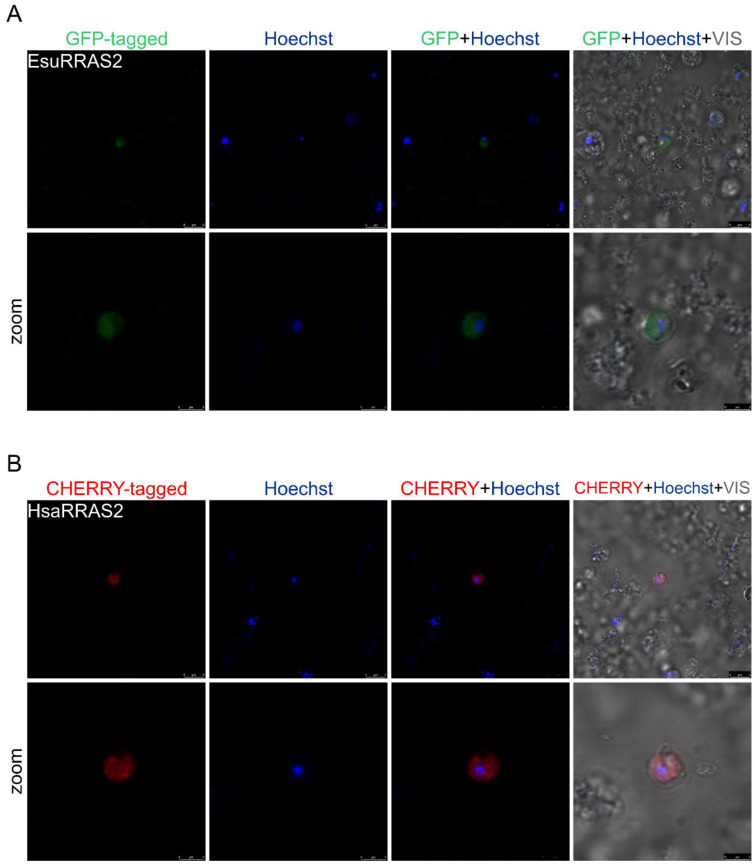
The human and sponge RRAS2 homologs do not localize in the nuclei of sponge cells. (**A**) Sponge RRAS2 was labeled with GFP, and (**B**) human RRAS2 was labeled with CHERRY. Cell nuclei were stained with Hoechst. Abbreviations: Esu, sponge *E. subterraneus*; Hsa, human.

The sponge (Figure 9A) and human (Figure 9B) RRAS2 proteins did not colocalize with the Hoechst stain, implying that RRAS2 does not enter the sponge cell’s nucleus. Although we were not able to distinguish whether the RRAS2 proteins are localized in the cytosol or membranes due to the non-uniformity of the GFP and CHERRY signal intensity, we assumed that the RRAS2 proteins might also be localized in the cytosolic vesicles and not dispersed in the cytosol, per se (Figure 9). These results indicate a similar localization pattern of sponge and human RRAS2 proteins in the sponge and human cells.

### 2.3. Cytoplasmic Protein Localization

The developmentally regulated GTP-binding protein (DRG) subfamily consists of two paralogs, DRG1 and DRG2. Both are involved in protein translation [91], microtubule regulation [92], and cell proliferation [73,93]. DRG1 is an evolutionary conserved GTPase that has been implicated in various tumors, but its role in cancer is not yet fully understood. Our study [73], as well as other studies, confirmed the localization of DRG1 in the cytosol of various cell lines (MCF-7 and HeLa human cancer cell lines, mouse 3T3 cells, and *Drosophila melanogaster* cells) [94,95,96].

**Figure 10:**
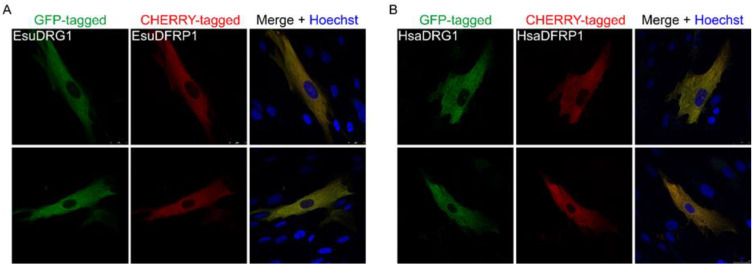
The (**A**) sponge and (**B**) human DRG1 homologs localize in the cytosol of human fibroblasts, MJ90. (**A**) Sponge and (**B**) human DRG1 were labeled with GFP and (**A**) sponge and (**B**) human DFRP1 with CHERRY. Colocalization of DRG1 with DFRP1 is visible in yellow. Cell nuclei were stained with the Hoechst. Abbreviations: Esu, sponge *E. subterraneus*; Hsa, human.

The expression of the human DRG1 protein requires the DFRP1 protein for its stabilization and localization in the cytosol of human cancer cells MCF-7 and HeLa [73,92]. Therefore, to determine the localization of the DRG1 protein, cDNAs for the sponge and human proteins were cloned into a vector containing the GFP marker, while the sponge and human DFRP1 were cloned into a vector containing the CHERRY reporter. We noticed the complete colocalization of the sponge DRG1 with the corresponding DFRP1 (Figure 10A) and the human DRG1 with the human DFRP1 in the cytosolic area (Figure 10B), without any obvious staining of the nucleus of MJ90 human fibroblast cells (Figure 10).

**Figure 11:**
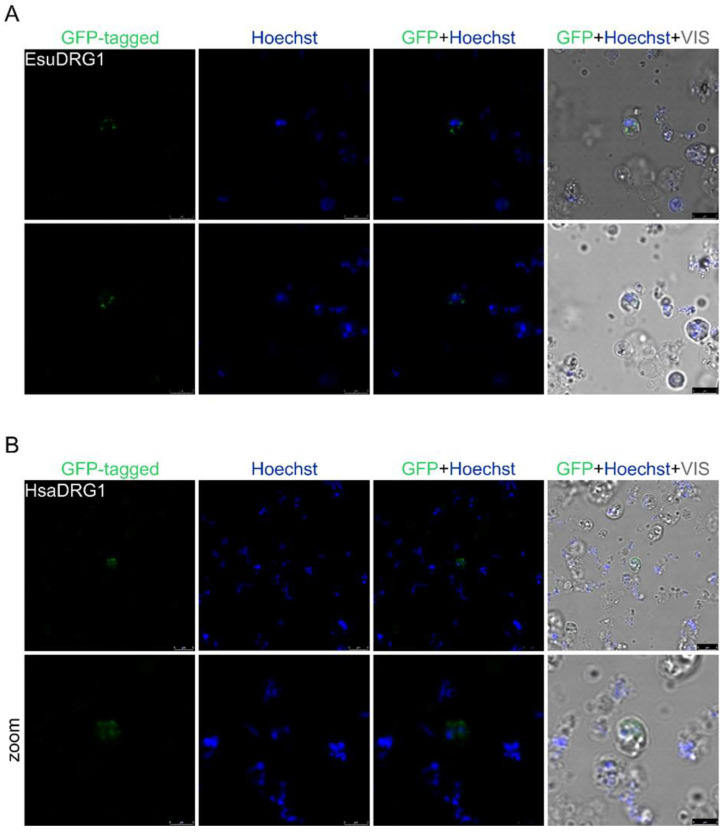
The human and sponge DRG1 homologs localize outside the nuclei of the sponge cells. (**A**) Sponge and (**B**) human DRG1 were labeled with GFP. Cell nuclei were stained with Hoechst. Abbreviations: Esu, sponge *E. subterraneus*; Hsa, human.

To determine the DRG1 protein localization in sponge cells, the sponge and human cDNAs were cloned into a vector with GFP. However, due to the high red autofluorescence of sponge cells, we cloned the sponge and human DFRP1 cDNAs into a vector containing the MYC tag (not shown). We observed that the DRG1 protein localizes outside the nucleus. The morphology of the fluorescent signal suggests the localization of the exogenous sponge protein DRG1 in the sponge cell granules (Figure 11A). Similar results were obtained with the human DRG1 in the sponge cells (Figure 11B).

## 3. Discussion

To the best of our knowledge, currently, there is no optimized protocol for the transfection of cultured sponge cells or tissue. Since there are no commercially available sponge cell lines, the first choice when studying the exogenous protein expression in sponges is the transfection of the primary suspension cell culture or tissue. Dissociated sponge cells have the tendency to reaggregate and form a functional sponge again [97]. Primmorphs, reaggregates of dissociated cells, have been described for *S. domuncula*. However, these structures do not show the typical morphology of an adult specimen [98], limiting the extent of biological features that can be studied. To date, transfections of *Haliclona* primmorphs [51] and *S. domuncula* slice explants [52] have been described. Pfannkuchen and Brummer (2009) [56] transfected the gemmules of the sponge *S. lacustris*, while Rocher et al. transfected the buds of *O. lobularis* [55]. Sponge cell transfection has the largest chance of success if performed on dividing and metabolically active cells [99]. Therefore, we chose to perform our localization studies on freshly dissociated cells before their reaggregation into primmorphs and similar to a previously published study on the primary cell cultures of *Axinella corrugata* [99]. While Pfannkuchen and Brümmer (2009) [56] used particle bombardment to deliver the foreign plasmid DNA, Grasela et al. (2012), Schippers (2013), and Rocher et al. (2020) [51,55,99] used different lipid-based chemicals [100], and Revilla-i-Domingo et al. [52] used linear polyethyleneimine (jetPEI) for the introduction of foreign DNA into the sponges.

In this study, we have used several commercially available transfection reagents for the transfection of the sponge cells: Lipofectamine 2000 and Lipofectamine 3000, TurboFect, TurboFect in vivo, and DharmaFECT, as well as calcium chloride or saponin. Similar to previous transfection attempts on sponges, our experiments showed that the choice of transfection reagent does not affect the transfection efficiency. In the lipofection experiments, we replaced the Opti-MEM™ medium with fresh cave water, as we noticed that Opti-MEM™ is toxic to sponge cells, as already shown for sponge buds [55].

In order to achieve the attachment and adhesion of sponge cells that normally grow in suspension, we used several compounds for coating the bottom of the Petri dish—poly-L-lysine, laminin, and fibronectin. We also tried to fix the sponge cells with the most commonly used fixation reagent (paraformaldehyde or a paraformaldehyde/glutaraldehyde). However, we were not able to induce the sponge cells to adhere to the coated Petri dishes.

One of the important decisions while establishing a transfection protocol is the choice of a promotor. Previous studies indicate that the CMV (cytomegalovirus) promoter, which is widely used in mammalian cells, is also functional in the freshwater sponges *E. fluviatilis* [99] and *S. lacustris* [56], while the MPSV (myeloproliferative sarcoma virus) promoter is effective in the marine sponge *A. corrugata* [99]. Based on this, we did not additionally test the efficacy of different promotors. Furthermore, for our transfection and localization studies, we have used a reporter gene that encodes for a green fluorescent protein (GFP) used in Hydra [101] and in similar studies [52].

**Figure A1:**
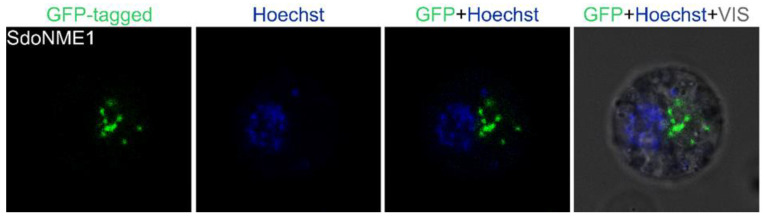
The sponge NME1 localizes outside the nuclei of the sponge cell. The sponge NME1 was labeled with GFP. Cell nuclei were stained with Hoechst.

**Figure A2:**
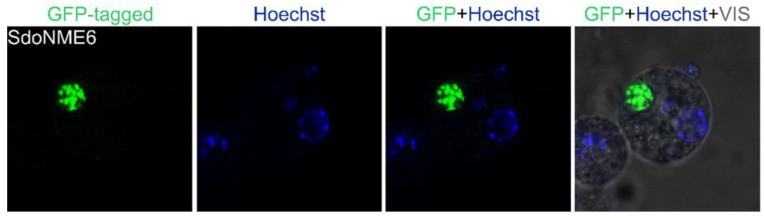
The sponge NME6 displays a dense fluorescent signal in the sponge cell outside the nucleus. The sponge NME6 was labeled with GFP. Cell nuclei were stained with Hoechst.

In our previous studies, we chose sponge and human cancer-related homologs with an already determined localization in human cancer cells, namely NME1 [70,102,103]) and NME6 [104,105,106]. The human NME1 is found in the cytosol and in the nucleus of HeLa cells [103,106], similar to our previous study of its homolog from the marine sponge *S. domuncula* [70]. NME6 is a ubiquitously expressed protein localized in the mitochondrial matrix, possibly associated with the mitochondrial inner membrane of human cells [104]. Interestingly, our studies [105] showed that the NME6 homolog from the sponge *S. domuncula* does not localize in the mitochondria of human cells but colocalizes with early, late, and recycling endosomes in human HeLa cells. To study the localization of these proteins in sponge cells, we attempted to introduce cDNAs from the marine sponge *S. domuncula’s* NME1 and NME6 into the dissociated sponge cells. However, we could not obtain a single viable *S. domuncula* cell expressing NME1 or NME6 after transfection. Therefore, we attempted to transfect the cells of another sponge species, *E. subterraneus*, with the same constructs, and we succeeded in obtaining a fluorescent signal (Appendix A, Figure A1 and Figure A2). The sponge NME1 localizes outside the nucleus, presumably in the sponge granules (Figure A1). The sponge NME6 also localizes outside the nucleus. The dense fluorescent signal indicates its localization in one of the organelles, possibly in the mitochondria (Figure A2). Based on these preliminary results, we continued the experiments presented herein using cDNA from *E. subterraneus*, and all subsequent transfections were performed on this sponge species. Although we encountered the expected difficulties in determining the protein localization due to low transfection efficiency, our results show that it is possible to transfect sponge cells in cultures. We were able to determine the localization of the proteins in the nuclei, cytosols, and possibly the membranes of the transfected sponge cells. Also, we showed that the localization of the sponge and human homologs in human tumor and normal cells is identical to their localization in the sponge cells. For example, sponge and human MYC localize in the nucleus, while DRG1 is localized outside the nucleus, i.e., in the cytosol. The evolutionary conservation of the intracellular localization of proteins from the sponge to humans points to the conservation of their biological function.

Furthermore, it is known that simple non-bilaterians, especially sponges, produce bioactive compounds, some of which have antibiotic, antiviral, anti-inflammatory, and antitumor activity [107,108]. A major obstacle to isolating and producing novel compounds is the limited availability of sponge material for preclinical and clinical testing [51,109,110]. Previous attempts to establish an immortalized, continuously dividing sponge cell line in cultures were hampered by numerous experimental problems [54,111]. Therefore, more effort is needed to develop sponge cell cultures, as well as transfection protocols, for sponges. Many issues need to be resolved to achieve higher expression levels of exogenous proteins and reproducible experiments and studies. This includes substantial improvements in sponge cell adhesion and fixation methodology, the discovery and application of specific sponge promoters, and advances in transfection reagents/buffers/media, appropriate both for sponge growth and transfection conditions.

## 4. Materials and Methods

### 4.1. Sequence Analysis

Homologs of human MYC and RRAS2 were found in our unpublished transcriptome of sponge *E. subterraneus* and identified in other sponge genomes at the Nacional Center for Biotechnology Information database (NCBI) using the blastp algorithm [112]. Protein sequences from sponge and human homologs were aligned using ClustalX 2.0 [81]. Aligned sequences were visualized using ESPript 3.0 [82], with indicated conserved domains adapted from the Conserved Domain database (NCBI) and additionally confirmed by the literature.

### 4.2. Cell Culture

Human cervical cancer cells, HeLa (ATCC cat. no. CCL-2), were maintained in Dulbecco’s Modified Eagle Medium with high glucose (DMEM, Sigma-Aldrich, St. Louis, MI, USA) supplemented with 10% fetal bovine serum (FBS, Capricorn Scientific, Ebsdorfergrund, Germany), 1% nonessential amino acids (Sigma-Aldrich), and a 1% antibiotic/antimycotic solution (Capricorn Scientific) in the humidified chamber at 37 °C and supplied with 5% CO_2_.

The normal neonatal human diploid fibroblast strain MJ90 was kindly provided by Dr. Olivia M. Pereira-Smith (the University of Texas, Health Science Center, San Antonio, TX, USA). MJ90 cells were cultured in high glucose DMEM (Sigma-Aldrich) supplemented with heat-inactivated 10% fetal bovine serum (FBS, Capricorn Scientific) and a 1% antibiotic/antimycotic solution (Capricorn Scientific) at 37 °C with 5% CO_2_.

The Ogulin cave sponge, *Eunapius subterraneus*, was collected from the Tounjčica cave at the Tounj location near Ogulin, Croatia. The sponge was maintained in cave water, transported to the laboratory, and placed in an incubator at 8 °C with occasional aeration. Sponge cell cultures were prepared by the adapted method for mechanical cell dissociation [113]. Briefly, the sponge was cleaned and homogenized using a manual homogenizer, followed by the separation of sponge cells through 200 µm, 100 µm, and 40 µm of mesh nylon to eliminate spicules and pieces of skeleton. The mesh was additionally washed with filtered cave water, and the filtered cells were allowed to settle at the bottom of the Falcon 50 mL tube for about 30 min at 4 °C. After the cells settled, the turbid supernatant was carefully removed, and new sterile cave water was added. The procedure was repeated until the complete clearance of the supernatant. Finally, the supernatant was removed with a pipette, and the precipitate containing the cells was transferred to a sterile tube. The cells were diluted in 500 µL of fresh cave water, and 1 × 10^6^ cells were seeded in Petri dishes divided into 4 wells with a coverslip on the bottom (4-chamber 35 mm glass bottom dish with a 20 mm microwell, Cellvis, D35C4-20-1.5-N).

### 4.3. Plasmids

For transfection experiments, our unpublished transcriptome of *E. subterraneus* was searched for homologs of human MYC and RRAS2. Identified sponge sequences were used to design primers for the amplification of the sponge MYC (EsuMYC) and RRAS2 (EsuRRAS2) from the cDNA library. EsuMYC and EsuRRAS2 were amplified, sequenced, and cloned into pEGFP-N1 and pEGFP-C1 vectors, respectively. The cDNA sequences of the human MYC (HsaMYC) and RRAS2 (HsaRRAS2) from commercially available plasmids were cloned into pEGFP-N1 and pmCherry-C1 vectors, respectively. Primers and restriction enzymes used for the cloning of EsuMYC, HsaMYC, EsuRRAS2, and HsaRRAS2 are listed in Table 1. The resulting constructs for the analysis of MYC and RRAS2 intracellular localization are GFP- or CHERRY-tagged. Other plasmids (EsuDRG1-GFP, HsaDRG1-GFP, EsuDFRP1-CHERRY, HsaDFRP1-CHERRY, EsuDFRP1-MYC, and HsaDFRP1-MYC) were already published [73].

### 4.4. Transfection

Chemical transfection: For lipid-based transfection (lipofection), HeLa cells (2 × 10^4^ cells/well) and MJ90 cells (7.5–9 × 10^4^ cells/well) were seeded on a sterile glass coverslip in a 24-well plate to achieve ~80–90% confluence. After 24 h, cells were transfected with plasmids of interest using Lipofectamine 3000 (Thermo Fisher Scientific, Waltham, MA, USA) according to the manufacturer’s protocol and incubated at 37 °C for an additional 24 h. Turbofect in vivo (Thermo Fisher Scientific, R0533) was used for sponge cell transfection according to the manufacturer’s instructions for in vivo transfections. Sponge cells were transfected with selected vectors, after which they were incubated for 24 h at 8 °C.

Electroporation: MJ90 cells were passaged 1 day before transfection to achieve 50–70% confluency on the day of the experiment. Before electroporation, cells were washed with a PBS and counted. A total of 1 × 10^7^ cells were resuspended in 0.4 mL Opti-MEM (Invitrogen), and 20 μg of plasmid DNA was added to the cells and gently mixed. The cell/DNA mixture was transferred to a 0.4 mm gap cold electroporation cuvette and electroporated under the following conditions: 220 V, 950 μF, and 30 msec. About 1 × 10^5^ of the electroporated cells were transferred into 24-well tissue culture plates with coverslips and incubated in supplemented growth media for 24 h at 37 °C.

### 4.5. Detection of Fluorescent Proteins and Confocal Microscopy

Immunocytochemistry on HeLa and MJ90 cells was performed as previously described [114]. In short, the cells grown on coverslips were washed three times in the PBS and fixed with 4% sucrose/paraformaldehyde for 15 min. Sponge cells were not fixed. Hoechst (Sigma-Aldrich) was used to counterstain nuclei. The staining conditions were as follows: HeLa and MJ90, 1 mg/mL for 10 min in the PBS at RT, and for the sponge cells, 35 µg/mL for 30 min in fresh cave water at 8 °C. Confocal images were acquired using a laser scanning confocal microscope, Leica TCS SP8 (Leica Microsystems, Wetzlar, Germany). Additional image processing was performed by ImageJ software (National Institutes of Health, Bethesda, MD, USA) and Adobe Photoshop 2020 (Adobe Systems Incorporated, Mountain View, CA, USA).

## 5. Conclusions

Sponges and their symbionts are often studied as a source of bioactive compounds with therapeutic potential. However, due to their position on the phylogenetic tree, they can also provide fundamental insights into the origin of animals and their diseases. Many human diseases, including cancer, have a genetic background. We now know that numerous disease-related genes were already present in the earliest animals and have homologs in sponges. Studying these genes from an evolutionary perspective is a novel approach that can offer additional insight into the disease-related gene properties, functions, and their roles in disease development. Herein, we show that the subcellular localization of the studied proteins is conserved from sponges to humans. In order to study protein localization, we developed a transfection protocol for sponge cells. Further improvements in transfection and other cell and molecular biology techniques applied to sponge cells are needed to achieve higher expression levels of exogenous proteins, more reproducible studies, and the sustainable use of sponges in biotechnology.

## Figures and Tables

**Table 1:** List of primers and constructs used in the study.

Construct Name/Organism	Origin	Cloned In	Primers/Restriction Site
EsuMYC-GFP *E. subterraneus*	Esu cDNA	pEGFP-N1	XhoI 5′-GTCTAGCTCGAGATGGCGTCGTTGGTAGAGTTC-3′ BamHI 5′-CTAGACGAATTCCGGGAAAAACTTTGCAGAAACTTC-3′
HsaMYC-GFP *H. sapiens*	HG11346-UT SinoBiological	pEGFP-N1	NdeI 5′-GTCTAGGAATTCATGCCCCTCAACGTTAGC-3′ BamHI 5′-CTAGACGGATCCGCGGACGCACAAGAGTTCCG-3′
EsuRRAS-GFP *E. subterraneus*	Esu cDNA	pEGFP-C1	XhoI 5′-GTCTAGCTCGAGGCATGGCGGCCAACAAAGAC-3′ BamHI 5′-CTAGACGGATCCTCACAGAATTACACATTTCTTC-3′
HsaRRAS2-CHERRY *H. sapiens*	RC204591 OriGene	pmCherry-C1	XhoI 5′-GTCTAGCTCGAGGCATGGCCGCGGCCGGCTGGCG-3′ BamHI 5′-CTAGACGGATCCTTAGAAAATGACACAATGGCAG-3′

## Data Availability

All the data generated or analyzed during this study are included in this published article.
